# Physician nurse care: A new use of UMLS to measure professional contribution: Are we talking about the same patient a new graph matching algorithm?

**DOI:** 10.1016/j.ijmedinf.2018.02.002

**Published:** 2018-02-09

**Authors:** Andrew D. Boyd, Karen Dunn Lopez, Camillo Lugaresi, Tamara Macieira, Vanessa Sousa, Sabita Acharya, Abhinaya Balasubramanian, Khawllah Roussi, Gail M. Keenan, Yves A. Lussier, Jianrong ‘John’ Li, Michel Burton, Barbara Di Eugenio

**Affiliations:** a Department of Biomedical and Health Information Sciences, College of Applied Health Sciences, University of Illinois at Chicago, 1919 W Taylor St., Chicago, IL 60612, United States; b Department of Health System Science, College of Nursing, University of Illinois at Chicago, 845 South Damen Ave, Chicago, IL 60612, United States; c Department of Computer Science, College of Engineering, University of Illinois at Chicago, 851 South Morgan Street, Chicago, IL 60607, United States; d Department of Health Care Environments and Systems, College of Nursing, University of Florida, PO Box 100187, Gainesville, FL 32610, United States; e Department of Medicine, College of Medicine, University of Arizona, 1501 N. Campbell Dr, Tucson, AZ 85724, United States; f The University of Arizona Health Sciences Center, 1295 North Martin Ave, Tucson, AZ 85721, United States

**Keywords:** UMLS, Physician documentation, Nursing documentation, Professional discontinuity, Health informatics

## Abstract

**Background:**

Physician and nurses have worked together for generations; however, their language and training are vastly different; comparing and contrasting their work and their joint impact on patient outcomes is difficult in light of this difference. At the same time, the EHR only includes the physician perspective via the physician-authored discharge summary, but not nurse documentation. Prior research in this area has focused on collaboration and the usage of similar terminology.

**Objective:**

The objective of the study is to gain insight into interprofessional care by developing a computational metric to identify similarities, related concepts and differences in physician and nurse work.

**Methods:**

58 physician discharge summaries and the corresponding nurse plans of care were transformed into Unified Medical Language System (UMLS) Concept Unique Identifiers (CUIs). MedLEE, a Natural Language Processing (NLP) program, extracted “physician terms” from free-text physician summaries. The nursing plans of care were constructed using the HANDS^©^ nursing documentation software. HANDS^©^ utilizes structured terminologies: nursing diagnosis (NANDA-I), outcomes (NOC), and interventions (NIC) to create “nursing terms”. The physician’s and nurse’s terms were compared using the UMLS network for relatedness, overlaying the physician and nurse terms for comparison. Our overarching goal is to provide insight into the care, by innovatively applying graph algorithms to the UMLS network. We reveal the relationships between the care provided by each professional that is specific to the patient level.

**Results:**

We found that only 26% of patients had synonyms (identical UMLS CUIs) between the two professions’ documentation. On average, physicians’ discharge summaries contain 27 terms and nurses’ documentation, 18. Traversing the UMLS network, we found an average of 4 terms related (distance less than 2) between the professions, leaving most concepts as unrelated between nurse and physician care.

**Conclusion:**

Our hypothesis that physician’s and nurse’s practice domains are markedly different is supported by the preliminary, quantitative evidence we found. Leveraging the UMLS network and graph traversal algorithms, allows us to compare and contrast nursing and physician care on a single patient, enabling a more complete picture of patient care. We can differentiate professional contributions to patient outcomes and related and divergent concepts by each profession.

## 1. Introduction

Physicians and nurses are an integral care dyad for providing care and treatment to hospitalized patients [1,2]. Their roles are interdependent going back to ancient Hindu scripture [3]. Hospital physicians diagnose and plan medical treatments. Nurses take responsibility for the hands-on-care while exercising independent judgment, intervening, and continuously re-evaluating patients’ health status and response to medical treatments [4]. Given this interdependence, collaboration and synergy (the active assessment or provision of care for a patient health problem by both professions) is clearly important and as nurse–physician communication has been linked to improved patient outcomes [5–7]. Poor MD-RN communication is a common cause of adverse events [8].

### 1.1. Background

The concept of records for patients’ hospital care as a means of communication was first introduced in 1910 by Dr. Ernest Codman [9]. He noted a poor quality of care and absence of documentation, and asserted that all hospitalized patients have records of their histories, physical exams, medications and laboratory tests [9]. Physician documentations that met this standard, started out as case reports for didactic learning, but evolved to a loose narrative that was further constrained as concern for legal ramifications grew with additional reimbursement requirements [10–12]. Karolinska Hospital was one of the first hospitals to computerize the physician problem-oriented medical record [13,14]. However, physician discharge summaries do not reflect nursing care [15].

Earlier than physician standards, Florence Nightingale asserted the vital role of nurses in “recording” their condition for improving patient health [2]. Despite Nightingale’s assertion, nursing documentation was outside the patient’s medical records and written on a “Kardex” in the 1950s [16]. But its value was recognized around the world [17] as well as its complexities [18]. Nursing documentation has used multiple frameworks including unstructured narratives, structured narratives (e.g. SOAP notes) clinical pathways, and problem-based approaches [18,19]. Beginning in the 1970s, nurse researchers developed standardized terminologies for nursing diagnoses, interventions and outcomes [20,21]. Tastan and colleagues in a review noted that the NANDA-I (nursing diagnosis), NOC (patient outcomes), and NIC (nursing interventions) terminologies have the most empirical support (> 70% of studies) [21]. During this nurse terminology development, an unequivocal definition of nurse sensitive patient outcomes was studied [22]. These terminologies can represent nursing care [4,23].

While physician and nurse documentation have varied historical norms and frameworks, both are included in modern electronic health records. Given the asynchronous work of hospitals’ nurses and physicians [24], the patient’s health record is integral in planning and communicating. There has been long standing research on nurse physician communication and collaboration across the world [25–27], but there are important limitations of this research. Many are qualitative exploration [28–30] or surveys based on perceptions [27,31,32] that are often disparate between physicians and nurses. These studies focus on process and attitudes of providers instead patient outcomes and patient details [25,31]. Prior interprofessional quantitative work focused on higher level physician concepts (Diagnosis Related Groups) of documentation between professions [33]. Here we propose a more detailed method to compute a quantitative measure from clinical care documentation from both nurses and physicians that can link to patient specific outcomes. All of the care in the hospital is not documented in the physician discharge summary nor is all of the care documented in the nursing documentation. By understanding how the terms from the two documentation standards are similar and related, we hypothesize we can obtain a better understanding about where the intersection of care occurs to measure the individual and combined contributions of both professions. The study objective is to gain insight into inter-professional care by developing a computational metric to identify similarities, related concepts and differences in physician and nurse work. The computational metric works by applying a graph traversal algorithms that leverage the existing Unified Medical Language System (UMLS) network to study the synergistic nature of care.

## 2. Materials and method

### 2.1. Design

We used a non-experimental correlational design. This research project was approved by the University of Illinois Institutional Review Board as exempt (#2012-0823).

### 2.2. Setting

Data was collected from existing electronic health record (EHR) (Cerner Millennium) data from a single urban academic hospital.

### 2.3. Data sources

We used documentation from physicians and nurses for 58 de-identified unique patients discharged with a medical diagnosis of heart failure from a random sample of 8 year of discharges.

#### 2.3.1. Physician discharge summaries

**Fig. 1:**
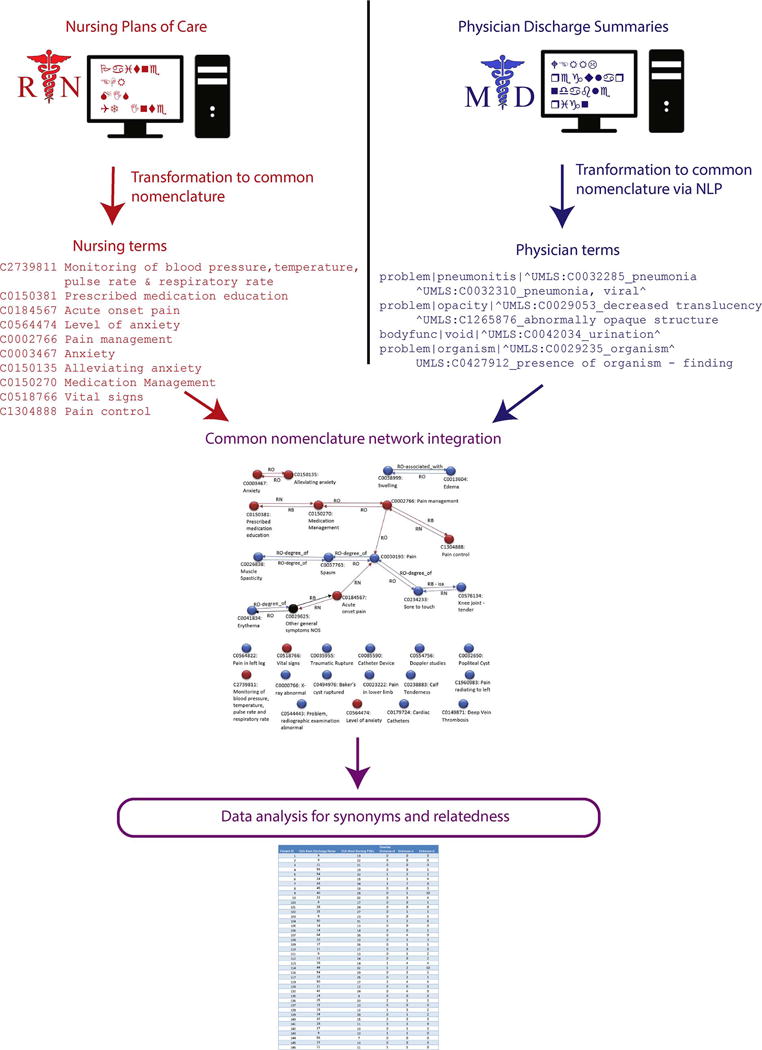
Overview of data analysis steps for the project. Physician and nurse documentation are transformed to compare them for synonymity and relatedness.

Previously, we processed eight years of physician summaries via the Medical Language Extraction and Encoding System (MedLEE) system [34,35], a Natural Language Processing (NLP) system. The NLP system produces a semi-structured output where concepts are mapped to the corresponding CUI (concept unique identifier) terms from the UMLS metathesaurus, a “common nomenclature” [36–38]. A random sample of 58 de-identified physician discharge summaries was selected. Summaries were created by physicians as free text or smart templates with additional free text. The entire dataset of physician summaries is called, “Physician Discharge Summaries”. The data derived via NLP, is called “Physician terms” (Fig. 1). The physician discharge summary includes several sections: history of present illness, medications list, and follow up appointments. We used the section labeled ‘hospital course’ as it relates most to the hospital care.

#### 2.3.2. Nursing plans-of-care

For this study in a hospital with minimal nursing documentation, two nursing informatics students created rich nursing plans-of-care (POCs), with the HANDS^©^ software, by retrieving cases similar to that described in the physician discharge summary from 40,661 HANDS^©^ POCs from a prior study in four Midwestern hospitals [23]. Also guiding the creation was patient’s numeric data (e.g. Vital signs) and patient’s brief free nursing free text documentation (discharge teaching complete) extracted from the EHR.

**Fig. 2:**
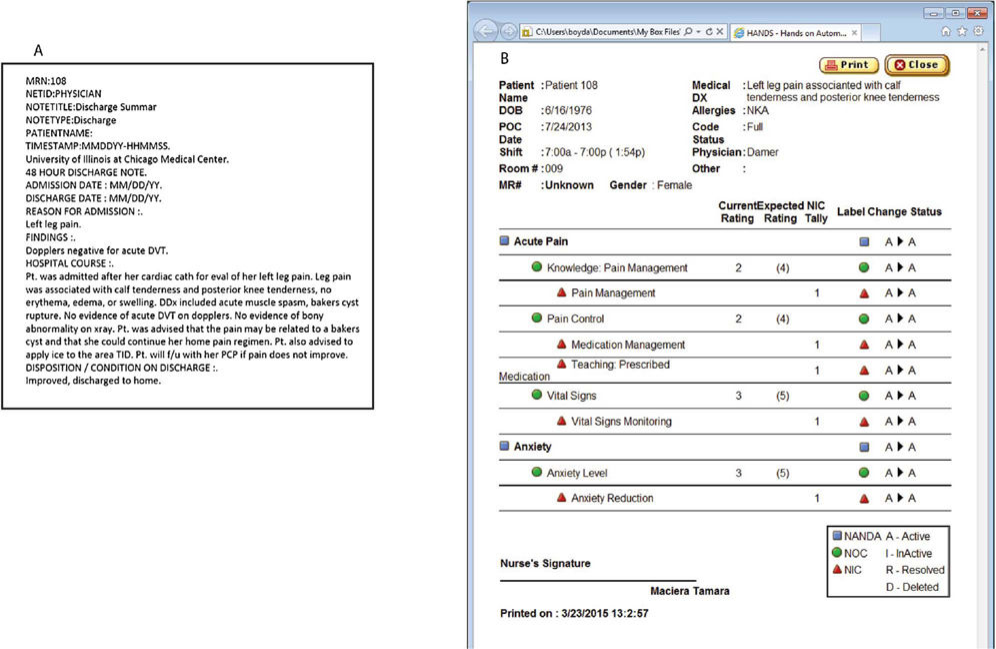
Excerpts from a physician discharge summary and a nurse plan of care.

HANDS^©^ is a nursing plan-of-care documentation software that represents nursing diagnoses, interventions, and outcomes respectively with NANDA-I, NIC, and NOC [20,39,40]. Nurses update the POCs at each shift change for non-flow sheet information (Fig. 2). A single hospitalization typically results in multiple POCs.

The student’s POCs were validated by two nurse authors with in depth knowledge of HANDS and nursing terminologies. Since the NANDA-I, NIC, and NOC have been incorporated into UMLS (Fig. 1), we were then able to leverage UMLS’s existing mapping to create a set of “Nursing terms”, identified by their CUIs from UMLS.

### 2.4. Analysis

The UMLS network enables relationships between the professions to be computed, visualized and analyzed. We compute both “synonymity” and “relatedness” to measure synergy.

#### 2.4.1. Synonyms

Synonyms represent exact matches between the nursing and physician controlled terms. A comparison per patient of the individual terms in the physician and nursing terms are examined for an exact match. For example in subject 7, the physician documented Dehydration, MedLEE mapped the term to UMLS C0011175: Dehydration. The nurse used the nursing terminology “Deficient Fluid Volume”, which is NANDA-I code 00027. The nursing term maps to the same UMLS term C0011175. Since the UMLS terms are identical, the two concepts are labeled synonyms, or atoms in UMLS terminology.

#### 2.4.2. Relatedness

**Fig. 3:**
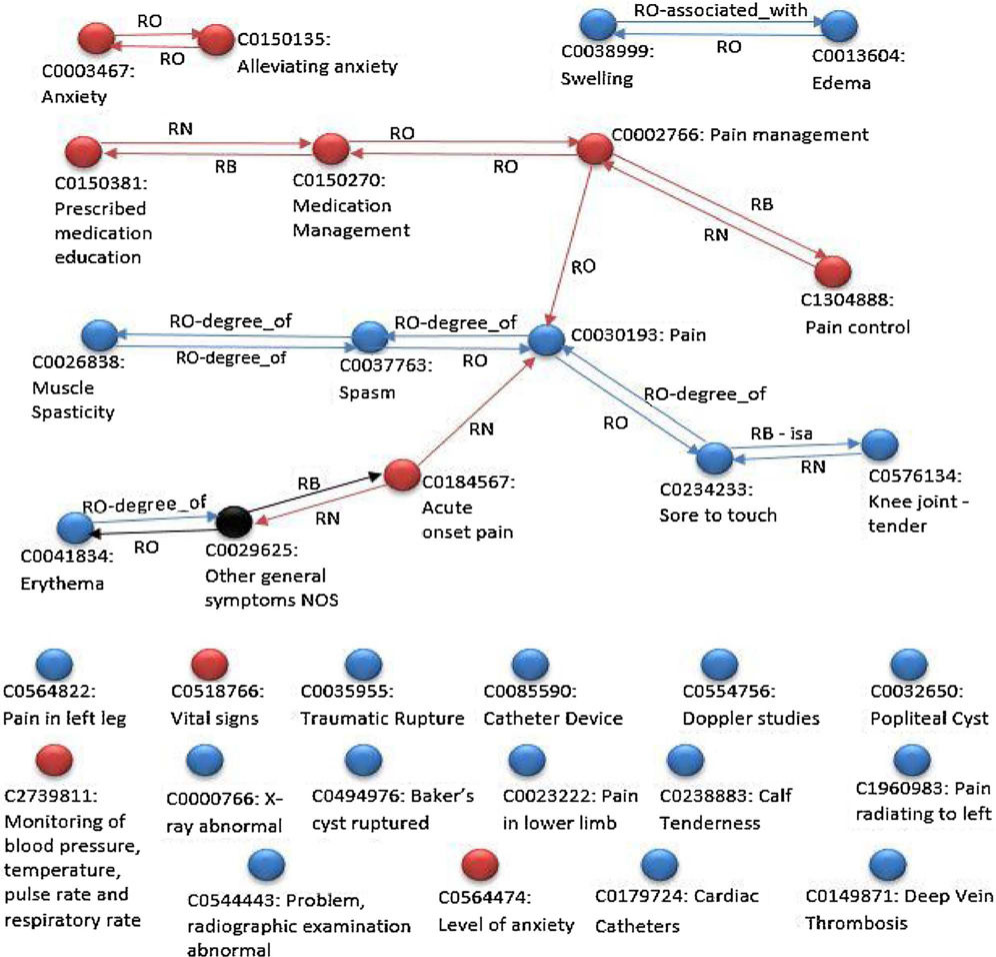
Sample network for research subject 108. Blue circles = MD terms. Red circles = nursing terms. Black circles = intermediate terms that connect the physician and nursing terms. Line = link (relationship) provided by the UMLS network.

Relatedness examines the non-identical relationship of terms. The common nomenclature (UMLS) links the terms together. A connection between two concepts is called a relationship (see Fig. 3). The relationships come from a variety of validated sources and are bidirectional [41]. If the relationship links between the terms are from a source vocabulary within the UMLS the source is documented [41]. If relationship links are from the UMLS metathesaurus or another service, the origin of the link is documented in the database. For example in Fig. 3, Alleviating anxiety is “otherwise” related to Anxiety (link RO), because it was created by the Metathesaurus at NLM. Other relationship links can be from any of the over 400 taxonomies and ontologies.

To reveal related concepts within a patient record, we construct a concept graph (Fig. 3). The inputs are a patient’s nursing terms and physician terms; we link the two by querying the UMLS Terminology Server. For example, ‘Pain’ (C0030193) was documented by the physician; however, the nurse documents ‘Pain Management’ (NIC 1400). The NIC code maps to the common nomenclature C0002766 ‘Pain Management’. ‘Pain’ (C0030193) and ‘Pain management’ (C0002766) are linked and related. We count distances in terms of relationships traversed; hence, the two terms are at distance 1, since one path links them.

At distance 2, physician terms and nursing terms may be linked through other concepts. For example, ‘Erythema’ (C0041834) was documented by the physician, whereas the nurse documented ‘Acute Pain’ (NANDA-I 00132). The NANDA-I code maps to the UMLS C0184567 ‘Acute Onset Pain’. Both concepts are linked to ‘Other General Symptoms NOS’ (C0029625). While ‘Other General Symptoms NOS’ was not documented, the concept links the two terms.

**Fig. 4:**
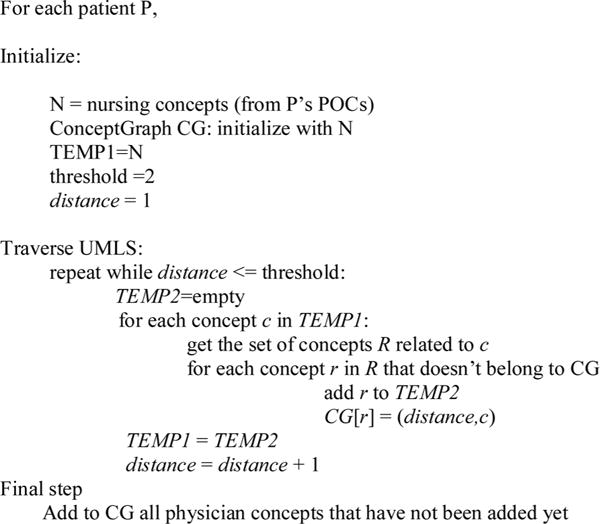
Algorithm to calculate the distance between terms.

In UMLS, 50% of terms (more than 1 million) are connected within a distance of 6 [42]. Deciding how many links to traverse in the network, is an empirical question; we have set the threshold to 2 to limit search time on UMLS servers. However, this is an adjustable parameter in the algorithm that traverses the UMLS network (see Fig. 4) We seed (start) the graph with an initial set of nursing terms, because on average there are fewer nursing terms [18] than physician terms [27]. The concept graph contains, for each concept c, a list of concepts related to it, with the distance between each pair of concepts.

At the end, for each concept reachable from the nursing concepts, the ConceptGraph (CG) in Fig. 4, records the shortest distance from a nursing concept, and the predecessor along that shortest path; these may or may not be physician concepts. The physician concepts that were not found by the algorithm are added as separate nodes (see isolated blue nodes in Fig. 3). The unconnected red nodes in Fig. 3 represent nursing concepts; these are the nursing nodes from which no connection has been found. Some physician and nursing terms are seen to be isolated because they either do not have any relation with other terms or are related through intermediate terms at distance greater than 2.

#### 2.4.3. Comparison

After creating the graphs for each hospitalization, the number of terms at a specific distance were compared between patients (see online table 2).

Using a Bayesian Calculator, a confidence interval was calculated for the number of patients with synonyms, where *X* is the total number of cases and *A* is the number of cases with synonyms.

$$Pr(A|X)=\frac{Pr(X|A)Pr(A)}{Pr(X|A)Pr(A)+Pr(X|\text{not}\;A)Pr(\text{not}\;A)}$$

#### 2.4.4. Manual inspection

A total of 10 of the patients’ records were selected and the “Hospital Course” section of the physician note and the HANDS notes were compared by one of the authors, to identify any content that this person thought was related (SA). The author who inspected the synonyms was not a health professional, as to limit bias of professional training in the results (see Table 1).

## 3. Results

The physician discharge summaries processed by NLP generated an average of 87 terms per summary. Of these 87 terms, an average of 27 terms were from the hospital course section, and were used as physician terms. A total of 945 distinct CUIs are in physician terms (see online table 2). The nursing POCs created within HANDS generated 18 terms on average. A total of 304 nursing terms were used. For each individual patient, a concept graph was generated (see Fig. 3).

### 3.1. Synonyms

The average number of synonyms (terms used in both physician and nurse documentation) between the two professions per patient hospitalization is 0.4 terms. For 26% of patients, synonyms existed between physician and nurse documentation (see online table 2). Using a Bayesian Calculator with a 95% confidence interval the range is 16–38% of patients whose records would include synonyms.

### 3.2. Relatedness

At a distance 1, an average of 1.2 terms were calculated. A distance of 1 means that each concept is directly linked to a concept derived by the other profession. At a distance 2, the number of terms is 2.6. On average, only 4 terms per patient are linked (at distance 1 or 2) between professions.

### 3.3. Visual

A visual analysis of the patient level hospital graphs revealed individual physician and nurse terms that do not link via UMLS (namely, the concept graph consists of several components that are not connected one to the other). For example “Deep Vein Thrombosis” (C0149871), from the perspective of a health care professional would consider the concept related to “Swelling” (C0038999) and “Acute onset pain” (C0184567) but they are far enough in the UMLS ontology that the algorithm does not link them.

### 3.4. Human

Results of the human evaluation of related concepts are in Table 1. The largest difference between the algorithm and the human was Pt 102, with 2 and 7 respectively. The average absolute difference is 1.9.

## 4. Discussion

We were able to leverage existing knowledge sources (UMLS) to evaluate the differences and similarities between physician and nurse care to create a more unified patient perspective. Taking advantage of the existing UMLS network, we took a knowledge driven approach of the professional differences between nursing and physician care at an individual patient level compared to prior research [33]. The proposed methodology is generalizable to multiple nursing terminologies, physician documentation, and different systems of nomenclature. We have used common elements of an electronic health record to allow others to conduct similar analysis. Unlike previous studies that have relied upon human judgment for relatedness of terms [43], the algorithm we propose measures the differences between physician and nurses using nonhuman links. Prior work also focused on a conceptual definitions of “Nurse-Sensitive” Patient Outcomes [22], however our analysis results in some overlap in terms between physician and nurse documentation (Fig. 3) which causes the evaluation of patient outcomes to be more complex and interdisciplinary sensitive. The prior work by van Beek et al. used Diagnose Behandeling Combinaties (DBC) (Dutch Diagnosis Related Groups DRG variant) and compared to nursing minimum dataset using HOMALs, a principal component analysis for nominal variables [33]. DRGs transform the complete hospital stay to a single disease grouping, with only one term per hospital stay. van Beek used only 23 unique DRG’s across all patients studied as well as 38 unique nursing terms across all patients [33]. In our study, we extracted on 945 unique terms from the hospital course for physicians and 304 unique terms for the nurse documentation, a significant increase in detail with fewer patients. Another difference in the two analyses is van Beek’s use of HOMALS [33] compared to the use of UMLS, an external knowledge domain. Through the use of UMLS, all relationships/linkages can be traced to prior knowledge with UMLS for additional analysis, where HOMALS can reveal relationships but not explain why.

To illustrate the insights from the new method, consider the single patient 108 (see Fig. 3), which is representative of an average patient case. There are no synonyms, there are two terms at distance 1, and one term at distance 2. In Fig. 3, the physician concept Pain (C0030193) is at distance 1 away from the nursing concept of Acute Onset of Pain (C0184567). The terms are related and refer to similar concepts. The nursing concept Acute Onset of Pain (C0184567) is two links away (distance 2) from the physician concept Erythema (C0041434). The concepts are related. By highlighting the differences, a more comprehensive picture of the patient is possible. The related concepts help to highlight the potential synergy between the professionals. The method of searching the UMLS network is not new (UTS Semantic Network Browser is a common example), but highlighting the professional source of the terms and leveraging their relationships is new. The relationships within the UMLS, are generated both through the source terminologies, as well as UMLS and other services. While lack of linkage does not prove that no relationship exists, UMLS provides the largest linkages of diverse health terms open to researchers worldwide to evaluate the interprofessional linkages. This is quantitative evidence for the hypothesis that physicians and nurses focus on different aspects of patient care and need integration for a complete picture.

Future use of this method to measure potential synergist care, as evidenced by use of related terms, could be critical to improving care quality in the temporally demanding hospital. From a patient safety perspective, during a root cause analysis (an in-depth analysis of the factors contributing to an unsafe environment) examining the professions scope and treatment can provide new insight. Additionally, by focusing on aggregate data from the individual wards, floors, and hospitals, treatment patterns could emerge and best practice guidelines could emerge to improve the quality of care. Finally, in this era of evidence based medicine, specific nursing interventions delivered and measured may offer an explanation of why medical treatments are effective in some patients but not others.

### 4.1. Common nomenclature (UMLS)

While the analysis has revealed similarities and differences between the languages of physician and nurse, the comparison of the terminologies is reliant upon the connections provided by the common nomenclature (UMLS). The common nomenclature is a well-studied clinical terminology relationship generator and health domain taxonomy [36–38]. The common nomenclature would need to create a large number of new concepts to encompass the paradigm of nursing [44]. In the 1990s, the Systematized Nomenclature of Medicine-Clinical Terms (SNOMED-CT)’s decided to include nursing terms, to account for the differences between the professions [45,46]. Evaluation of the nursing concepts in SNOMED continued for another decade [47,48]. Later research has evaluated the differences between nursing concepts within SNOMED-CT [49,50]. After years of work, nursing terminology was well integrated into the common nomenclature (UMLS).

The selection of the common nomenclature (UMLS) was deliberate. In considering networks, the common nomenclature (UMLS) is a highly connected network ideal for finding relatedness between professions. A different network, Systematized Nomenclature of Medicine-Clinical Terms (SNOMED-CT) [51,52], has relationships designed as an a-cyclical tree, which would enable easy comparison between two terms but would increase the distance between any two terms. The full network of UMLS is more representative of the complex nature of biomedicine.

### 4.2. Communication impact

Research examining multi-professional languages is rare. In Sweden, Terner et al. examined the documentation headings of eight professions in an electronic health record [53]. Slightly greater than 50% of the physician report headings (symptoms, medication) were used by nurses, and vice versa [53]. The headings are different than the discrete concepts, limiting comparison between the professions and our work. Another study from Norway shows how the terminologies for the hospital nurse and hospital physician are different but does not try to combine them at the individual patient level [54].

We also found more differences than similarities with on average only 4 related terms per patient. This was quite surprising to us given the high acuity and number of patient problems today’s hospitalized patient is likely to have. One concern that arises with the lack of shared terms is whether physician and nurses understand the subtle differences between the terminologies they use.

One could argue that nurses and physicians should be forced to use a common set of terms. However, each profession has a diversity of concepts and purposes. Asking either profession to only use terms that another profession would need, limits the care and would be a detriment. Each profession needs to reflect their comprehensive contribution to care. In addition, nursing and physician documentation is meant to be a reflection of their clinical assessments and activities. Additional research on the differences and similarities in their documentation should yield important insights about patient outcomes and hospital processes.

### 4.3. Limitations

One limitation of our study is that the nurses’ terms were not derived from actual nursing documentation, but developed in response to discharge summaries. An initial attempt evaluated the existing nursing documentation in our hospital. Unfortunately, the terms and concepts used in this hospital were very sparse. When narrative free text information was documented, the information was often quite vague (i.e. “Talked to patient about POC”), and did not provide sufficient information to evaluate the nursing care provided. Creating these POCs by hand strengthens the results as the similarity we have found between physician and nursing documentation is likely to be an upper bound, due to the fact the nursing documentation was generated taking into account the physician discharge summary. In practice this would not occur and the similarity between the two documentations would be even lower.

Another limitation of this methodology is the fact it is reliant upon the linkages of UMLS metathesaurus. In the human evaluation, we discovered the largest disagreement between humans and the algorithm in patient 102 were there is a disagreement of 5 terms. However, with an average absolute difference of 1.9 between the algorithm and human evaluators, the algorithm is an approximation of the collaboration between professions.

Another limitation is the number of patients analyzed. The 58 physician discharge summaries were randomly selected before deidentifying so the likelihood is that the discharge summaries reflect a diversity of physicians. The nursing POCs were created by two nursing students, and were then edited and audited by faculty who are experts in nursing terminology. Due to the above variables, the methodology will need to be applied to larger datasets for reproducibility.

Another limitation of this methodology is the potential for gaps in the Natural Language Processing creating false negative due to the limitations of the algorithms or missing concepts within UMLS. However, applying MedLEE to the University of Illinois hospital dataset revealed 24,826 distinct UMLS codes for a single year compared to 3271 distinct ICD-9-CM or CPT codes for the same hospital visits [55]. Potential false negatives due to lack of knowledge of terms or missing concepts will be present in both human and computer evaluation of interprofessional terminology.

The complex relationship between the nursing and physician terms is just beginning to be uncovered in this analysis. We use standard terminologies because they have properties that are stronger than free text: the desired features and defining themes of controlled vocabularies in healthcare called the “desiderata” have been studied and applied for more than 15 years providing strength to this work [56]. Another limitation of our work is that a single site, a single population, and a single country may not be representative of care documentation by all physicians and nurses across the world.

## 5. Conclusion

Currently, no formal metric exists to compare and contrast work between nurses and physicians. Norma Lang has stated, “If we cannot name it, we cannot control it, practice it, teach it, finance it, or put it into public policy” [57]. The benefit of this analysis is the potential for improving care by highlighting the differences between the two professionals’ care for a complete picture. Additional future analysis will need to compare the results of the qualitative exploration of synergistic care [27–29] to the quantitative metric developed in this study. The future of inter-professional care has a long road to become successful. Future directions include identifying the variability in synonym/relatedness between hospitals and analyzing the impact on patient outcomes such as readmissions to shed light on how potential synergistic care between nurses and physicians can improve outcomes.

## Supplementary Material

supplement

## Figures and Tables

**Table 1:** Table of related terms per patient for physician and nursing terms comparing human and UMLS curated lists for 10 patients.

Patient ID	Human curated	UMLS linked terms	Absolute difference
100	1	1	0
101	3	0	3
102	7	2	5
103	3	1	2
105	1	0	1
106	2	1	1
108	3	4	1
109	6	2	4
112	1	2	1
113	8	9	1

## Summary points

### Prior

- Physician nurse communication was previously studied in a qualitative manner.
- No prior studies used knowledge based algorithms to compare nurse physicians documentation.

### Study added

- Unified Medical Language System (UMLS) can be leverage to study professional differences.
- Through documentation in the electronic health record better evaluation of physician and nurse collaboration can evaluate systems of care.

## Appendix A. Supplementary data

Supplementary data associated with this article can be found, in the online version, at https://doi.org/10.1016/j.ijmedinf.2018.02.002.
