# Supplementary material for: Physician nurse care: A new use of UMLS to measure professional contribution: Are we talking about the same patient a new graph matching algorithm?
Source: Int J Med Inform. Author manuscript; Available in PMC 2018 May 1. (PMC5909845; doi:10.1016/j.ijmedinf.2018.02.002)
Supplement: supplement [file NIHMS958176-supplement.docx]

**Online Table 1 Abbreviations**

| Descriptions | Abbreviations |
| --- | --- |
| Physicians concepts about hospital course extracted out of discharge summaries via NLP | Physician terms |
| NANDA-I, NOC, and NIC terminology mapped to common nomenclature | Nursing terms |
| Unified Medical Language System Version 2014AA | UMLS |
| Concept Unique Identifiers | CUI |
| North American Nursing Diagnosis Association International | NANDA-I |
| Nursing Outcome Classification | NOC |
| Nursing Interventions Classification | NIC |
| American Nurse Association | ANA |
| Medical Language Extraction and Encoding System | MedLEE |
| Natural Language Processing | NLP |
| Electronic Health Record | EHR |
| Systematized Nomenclature of Medicine-Clinical Terms | SNOMED-CT |
| communication accommodation theory | CAT |
| Plans of Care | POC |
| UMLS CUI | Common nomenclature |

**Online Table 2. Table of synonyms and relatedness per patient for physician and nursing terms.**

| **Patient ID** | **CUIs from Discharge Notes** | **CUIs from Nursing POCs** | **Overlap Distance=0** | **Distance=1** | **Distance=2** |
| --- | --- | --- | --- | --- | --- |
| 1 | 9 | 19 | 0 | 0 | 0 |
| 2 | 9 | 22 | 0 | 0 | 0 |
| 3 | 11 | 21 | 0 | 0 | 0 |
| 4 | 55 | 19 | 0 | 0 | 1 |
| 5 | 54 | 33 | 1 | 2 | 2 |
| 6 | 24 | 18 | 1 | 1 | 4 |
| 7 | 69 | 34 | 3 | 7 | 9 |
| 8 | 45 | 18 | 0 | 0 | 3 |
| 9 | 40 | 28 | 0 | 1 | 10 |
| 10 | 22 | 32 | 0 | 3 | 4 |
| 100 | 8 | 17 | 0 | 0 | 1 |
| 101 | 28 | 24 | 0 | 0 | 0 |
| 102 | 25 | 27 | 0 | 1 | 1 |
| 103 | 8 | 13 | 0 | 0 | 1 |
| 104 | 90 | 31 | 1 | 2 | 8 |
| 105 | 14 | 13 | 0 | 0 | 0 |
| 106 | 14 | 14 | 0 | 0 | 1 |
| 107 | 64 | 38 | 0 | 4 | 9 |
| 108 | 20 | 10 | 0 | 1 | 3 |
| 109 | 17 | 26 | 0 | 1 | 1 |
| 110 | 11 | 17 | 0 | 0 | 0 |
| 111 | 9 | 13 | 0 | 1 | 2 |
| 112 | 13 | 24 | 0 | 0 | 2 |
| 113 | 39 | 14 | 1 | 4 | 4 |
| 114 | 44 | 32 | 1 | 2 | 10 |
| 116 | 54 | 29 | 0 | 3 | 5 |
| 117 | 15 | 25 | 0 | 2 | 1 |
| 119 | 50 | 17 | 3 | 4 | 4 |
| 128 | 21 | 12 | 0 | 0 | 0 |
| 132 | 42 | 24 | 0 | 4 | 9 |
| 135 | 14 | 9 | 0 | 0 | 0 |
| 136 | 25 | 20 | 2 | 1 | 3 |
| 137 | 15 | 10 | 0 | 0 | 0 |
| 138 | 18 | 12 | 1 | 3 | 2 |
| 139 | 24 | 38 | 0 | 1 | 2 |
| 140 | 37 | 15 | 2 | 3 | 3 |
| 141 | 28 | 11 | 3 | 3 | 9 |
| 142 | 27 | 10 | 0 | 1 | 3 |
| 143 | 9 | 10 | 1 | 1 | 0 |
| 144 | 56 | 7 | 0 | 0 | 0 |
| 145 | 23 | 10 | 0 | 0 | 0 |
| 146 | 11 | 11 | 1 | 1 | 0 |

| **Patient ID** | **CUIs from Discharge Notes** | **CUIs from Nursing POCs** | **Overlap Distance=0** | **Distance=1** | **Distance=2** |
| --- | --- | --- | --- | --- | --- |
| 147 | 49 | 8 | 1 | 3 | 7 |
| 148 | 5 | 6 | 0 | 1 | 2 |
| 149 | 59 | 19 | 0 | 1 | 4 |
| 150 | 1 | 13 | 0 | 1 | 0 |
| 151 | 9 | 25 | 0 | 1 | 0 |
| 152 | 3 | 9 | 0 | 0 | 0 |
| 153 | 19 | 10 | 0 | 1 | 3 |
| 154 | 2 | 9 | 0 | 0 | 0 |
| 155 | 40 | 14 | 0 | 0 | 1 |
| 156 | 14 | 20 | 0 | 0 | 0 |
| 157 | 26 | 10 | 0 | 1 | 1 |
| 158 | 6 | 17 | 0 | 1 | 1 |
| 159 | 37 | 10 | 1 | 0 | 8 |
| 160 | 13 | 14 | 0 | 0 | 0 |
| 161 | 33 | 21 | 0 | 0 | 2 |
| 162 | 15 | 12 | 0 | 0 | 4 |
| **Average** | **26.6** | **18** | 0.39 | 1.15 | 2.58 |
